# Supplementary material for: Solanum nigrum Fruit Extract Modulates Immune System Activity of Mealworm Beetle, Tenebrio molitor L
Source: Toxins (Basel). 2023 Jan 12;15(1):68. doi: 10.3390/toxins15010068 (PMC9861574; doi:10.3390/toxins15010068)
Supplement: Supplementary file 1 [file toxins-15-00068-s001.zip › toxins-2013237-supplementary.pdf]

Supplementary materials for: *Solanum nigrum* fruit extract modulates immune system activity of mealworm beetle, *Tenebrio molitor* L.

Table S1. Primers used in the study

Name	Forward primer	Reverse primer	References
**Cecropin**	ATGGACAACCAATGCCACCC	GGTCTTCGATTCCGTTGCCT	Jacobs et al. 2017
**Tenecin-3**	CATCACGACGGACATCTGGG	TAAATGTCCGCCTGGTTGGC	Jacobs et al. 2017
**Toll**	TGCGTAGCAAACAGGTGGAT	TCGCGTAGCGGTAGTAGAGA	Jacobs et al. 2017
**RPL13a**	TCGTCGTGAGATGCGAACAA	CTGCTTCCCACGTTCTGTCT	Jacobs et al. 2017

References

Jacobs, C.G., J.D. Gallagher, S.E. Evison, D.G. Heckel, A. Vilcinskas, and H. Vogel. 2017. Endogenous egg immune defenses in the yellow mealworm beetle (*Tenebrio molitor*). *Developmental & Comparative Immunology* 70:1-8.

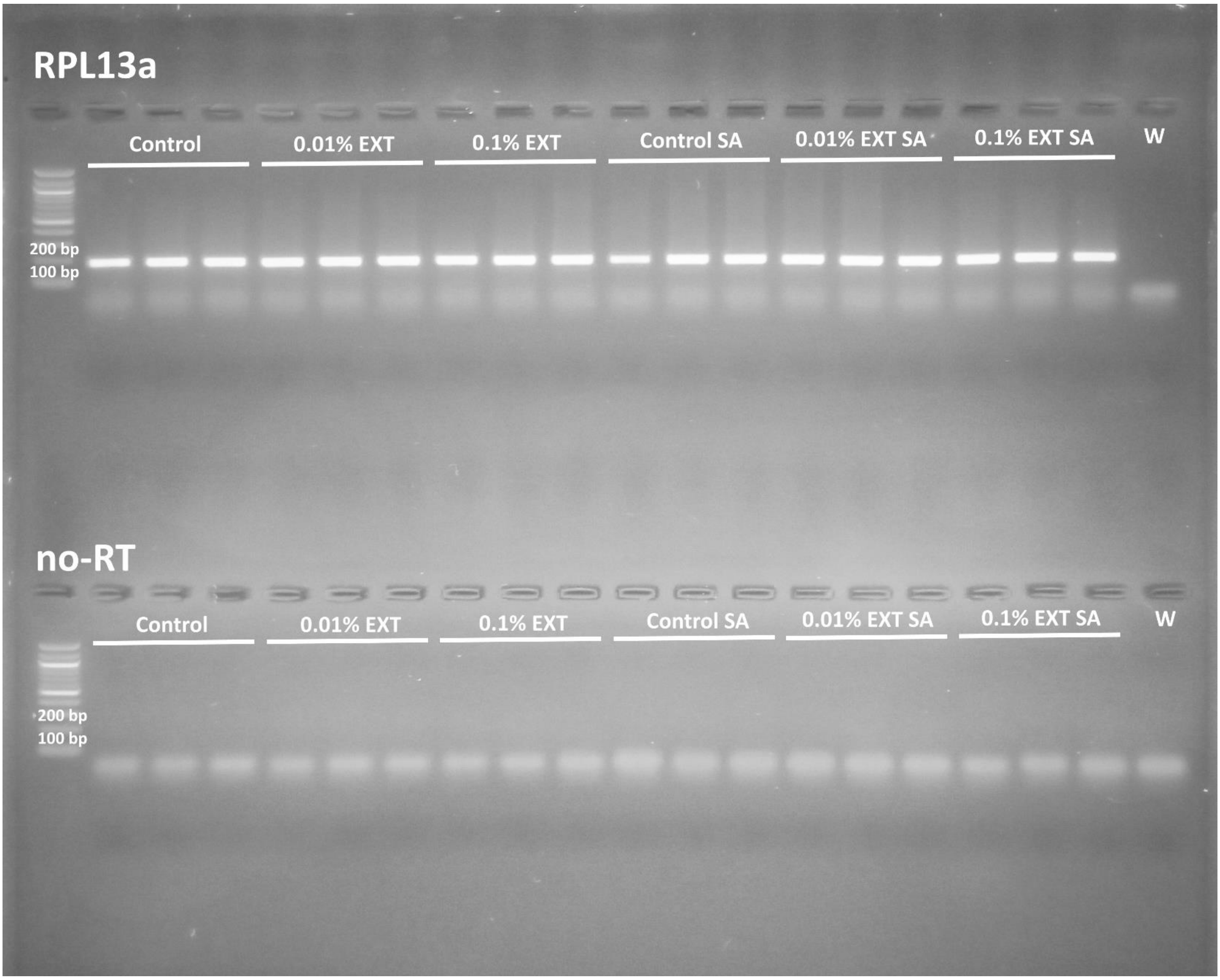

Figure S1. Positive and negative control for Reverse transcription quantitative PCR (RT-qPCR) analysis. The samples were checked using Reverse transcription PCR (RT PCR) method. Electrophoresis of RT PCR products was performed using a 2% TAE agarose gel stained with ethidium bromide. RPL13a - positive control of obtained samples; no-RT – negative control without using of reverse transcriptase during transcription of RNA to cDNA; Control – samples collected 24 hours after injection of physiological saline; 0.01% EXT – samples collected 24 hours after injection of physiological saline and *Solanum nigrum* fruit extract at concentration 0.01%; 0.1% EXT – samples collected 24 hours after injection of physiological saline and *Solanum nigrum* fruit extract at concentration 0.1%. Control SA – samples collected 24 hours after injection of physiological saline from additionally immunized beetles. 0.01% EXT SA– samples collected 24 hours after injection of physiological saline and *Solanum nigrum* fruit extract at concentration 0.01% from immunized beetles; 0.1% EXT SA– samples collected 24 hours after injection of physiological saline and *Solanum nigrum* fruit extract at concentration 0.1% from immunized beetles; W -water (no template control).
